# Towards a transparent and reproducible AI-assisted research paper writing

**DOI:** 10.1186/s44342-025-00057-0

**Published:** 2025-12-02

**Authors:** Jeongbin Park

**Affiliations:** School of Biomedical Convergence Engineering, Pusan National University, Yangsan, 50612 Republic of Korea

**Keywords:** AI-assisted writing, Scientific integrity, Transparency, Reproducibility, Language barriers

## Abstract

Artificial intelligence (AI)-assisted scientific writing is now a common practice in academic publishing, yet concerns persist regarding the authenticity and reproducibility of AI-generated content. While AI tools offer significant advantages, particularly for non-native English speakers who face substantial linguistic barriers in scientific communication, the risk of AI hallucinations and fabricated citations threatens the integrity of scholarly discourse. Journals often require disclosure of the entire AI prompt rather than meaningful intellectual contributions, but this is becoming increasingly impractical as AI prompts are getting longer and more complex. In this paper, I argue that transparency in AI-assisted writing should focus on capturing the author’s core research perspective and section-specific key points—the foundational elements that drive meaningful scientific communication. To address this challenge, I developed a web-based tool that implements a human-in-the-loop approach requiring authors to define their research perspective and create detailed outlines with key points before any AI text generation occurs. The tool mitigates AI hallucination by only allowing the use of user-provided citations and generating transparency reports documenting the key elements used for text generation. I validated this approach by writing this paper using the tool itself, demonstrating how the transparency reporting method works in practice. This methodology ensures that AI serves as a linguistic tool rather than a content generator, preserving scientific integrity while democratizing access to high-quality academic writing across linguistic and cultural boundaries.

**Supplementary Information:**

The online version contains supplementary material available at 10.1186/s44342-025-00057-0.

## Introduction

The submission of AI-assisted manuscripts to academic journals has become increasingly prevalent, with editors frequently encountering publications that demonstrate clear evidence of artificial intelligence involvement [3, 7]. While this phenomenon presents legitimate concerns regarding scientific integrity [1, 2, 4–6], the fundamental challenges that require attention are not the application of AI technology per se, but rather the maintenance of originality and reproducibility in scholarly communication. Primary concerns arise from AI hallucinations that may introduce fabricated citations and erroneous information into manuscripts, particularly in the absence of adequate human verification [2, 4].

However, complete prohibition of AI assistance may fail to recognize its substantial benefits, particularly for non-native English speakers who encounter significant linguistic barriers in scientific publishing [8, 9]. Many journals now allow AI use, but often require authors to disclose the full AI prompts used in manuscript preparation. But this is becoming increasingly impractical as AI prompts are getting longer and more complex, and as tools are getting more sophisticated. Rather than mandating comprehensive disclosure of increasingly complex AI prompts, I propose that such tools should be fully open-sourced, and their transparency mechanisms should focus on the fundamental elements that constitute meaningful scientific communication: the author’s core research perspective and section-specific key points derived from established scientific writing methodology.

Today, several commercial tools, such as *Scite* or *Paperpal*, have been developed for AI-assisted scientific writing. However, these tools are black-boxed; there is no way to understand how human originality is incorporated and how they mitigate hallucinations while generating text.

To this end, I present a structured web-based tool that implements a human-in-the-loop approach, which incorporates multiple AI-assisted steps, including perspective defining, outlining, and text drafting. This step-by-step nature of the tool promotes human interaction at each step, which enables authors to develop their research concepts transparently while utilizing AI’s linguistic capabilities, thereby preserving scientific integrity while facilitating equitable access to high-quality academic writing across linguistic boundaries. The tool is fully open-sourced along with all AI prompts.

## Results

**Fig. 1:**
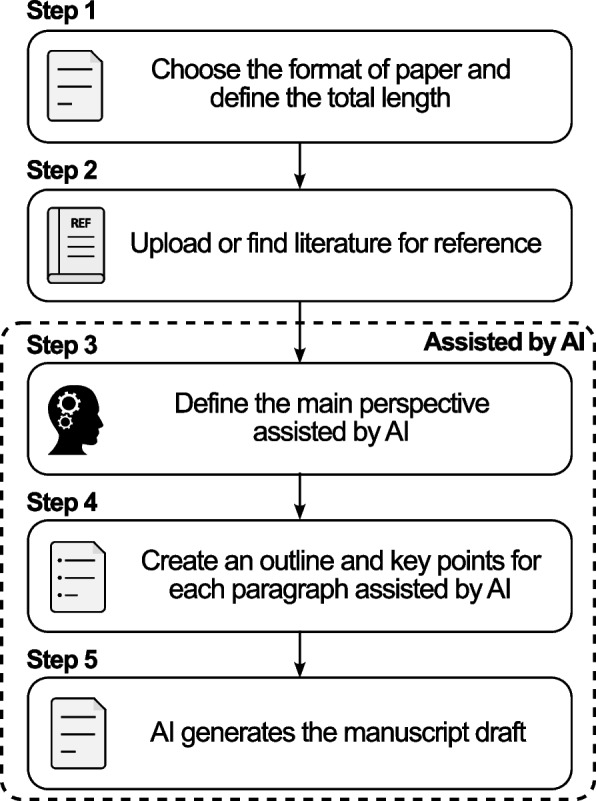
Workflow of the proposed web-based AI writing assistant. Steps 3–5 are AI-assisted processes for defining perspectives, outlining content, and generating the manuscript draft

The web-based tool works in five straightforward steps that build on how people already learn to write scientific papers (Fig. 1).

In the first step, authors choose the format of the paper and define the total length. In the second step, authors start by choosing the literature that they want to reference from their field. In the third step, authors write down their main perspective on the research topic in whatever language feels most comfortable to them, with an AI assistant to refine and clarify their ideas. In the fourth step, authors create an outline with key points for each section and paragraph, again working in their native language with AI assistance to structure their thoughts. Finally, AI generates an English manuscript based on these author-created perspectives and outlines using pre-written AI prompts.

Here, AI prompts were designed to include all user-provided materials and generated texts in the previous steps, such as paper references, perspectives, and key points, and include a declaration that the generated output should only be based on the user-provided materials to mitigate hallucinations. This strategy was validated using three test cases, which showed the high-quality text generation performance of the tool compared to two other commercial tools (see Supplementary material). This approach is similar to retrieval-augmented generation (RAG) in that it provides additional contextual information to LLMs for reference; however, it has been reported that RAG cannot eliminate hallucinations [10]. Instead, what makes this approach work is that authors stay involved at every step. Authors can review what the system produces and make changes whenever the output does not match what they intended.

After writing, the web tool generates a "transparency report". This document contains the author’s perspective and key points that were used to generate the text. By reading the transparency report, editors and reviewers can then see exactly what ideas drove the research.

The tool was fully open-sourced, with the prompts available as part of the source code. Therefore, the transparency report, combined with publicly available AI prompts, also provides the complete AI prompts that were used to generate the paper, as many journals require today.

The tool works entirely within the client-side web browser without a backend server, which means all user data remains on their local machines rather than being transmitted and saved within remote servers. This design enhances security by keeping sensitive research content under the author’s direct control. For researchers working with particularly sensitive material or those in institutions with strict data security requirements, the tool also has an option to run AI models locally on their own hardware, providing an additional layer of privacy protection. Finally, the tool was packaged using Docker, making it easy to deploy in any computing environment—even allowing the deployed tool to be accessed without an internet connection with a local LLM.

## Discussion

The main challenge in AI-assisted scientific writing lies in establishing the appropriate boundaries between tool usage and human oversight while preserving originality and academic responsibility. This work proposes that maintaining originality requires deliberate human intellectual investment at critical decision points—defining research perspectives, curating literature, and structuring arguments—rather than merely reviewing AI outputs post-generation. The structured workflow presented here enforces this by requiring authors to articulate their contributions explicitly before AI assistance occurs, creating clear delineations between human intellectual work and AI linguistic processing. This approach addresses concerns about diminished originality while maintaining the practical benefits of AI assistance for researchers navigating complex academic writing demands.

This tool represents a starting point for addressing current challenges in AI-assisted academic writing, acknowledging that nowadays we cannot prevent researchers from using AI to generate papers, but we can work toward more transparent and responsible implementation. However, this tool may also introduce potential negative effects, including creating new dependencies on AI services that could disadvantage researchers in resource-constrained environments, or potentially leading to the homogenization of writing styles that might reduce diversity in academic discourse. But as this tool is open-sourced, community-driven development may address these limitations through collaborative improvement over time, by incorporating more copyright-free texts as writing examples.

The tool is freely available at https://research.pnucolab.com, with complete source code accessible at https://github.com/pnucolab/paper-writing-assistant.

## Methods

### Implementation and data security

The tool was built using SvelteKit with TypeScript that runs entirely in the user's browser. The tool was designed to keep all user data on their local machines, with no server-side data storage. User content was transmitted only to their chosen AI providers through API connections. The tool was integrated with multiple language models through the OpenRouter API including popular AI models such as GPT, Claude, and Gemini. Support for custom OpenAI-like API endpoints was also added to allow users to connect to locally hosted models if needed. However, LLMs with large parameters are generally recommended for high-quality text generation. Thus, a high-performance GPU server is recommended to ensure model performance and low latency.

### Prompt engineering

To avoid the AI model from writing overly complex text, the AI prompts were customized using writing samples from the author’s PhD dissertation as a style reference [11], as it was free from copyright issues and written in typical academic writing standards. This customization helped produce clearer, more accessible text while maintaining scientific accuracy. The AI prompts were designed to explicitly prohibit generating fake references or data, requiring the models to work only with information provided by users.

### Transparency reporting

The tool was programmed to automatically generate transparency reports that documented the key elements used to generate text. These reports captured model information, section outlines, and key points that guided text generation. This documentation is to help editors and reviewers easily understand the key ideas underlying the generated text. Also, as the source code of this web tool and the prompt are open-sourced, together they provide a complete AI prompt used for text generation.

### Real-world application

This paper was written using the web tool described herein to test and validate the approach. The process began with selecting relevant literature on AI-assisted scientific writing and entering citations into the tool. The core perspective on transparent AI use in academic publishing was then developed, and key points for each section were outlined in the native language. English text was generated by the tool based on these inputs, which were then revised and edited by the author. A transparency report was generated to demonstrate how to disclose the report in practice (available as supplementary material).

## Supplementary Information

Supplementary Material 1. AI-assisted Writing Transparency Report.

Supplementary Material 2. Example output comparison with other tools.

## Data Availability

No datasets were generated or analysed during the current study.
