# Correction: Towards a transparent and reproducible AI-assisted research paper writing

**DOI:** 10.1186/s44342-025-00062-3

**Published:** 2026-04-17

**Authors:** Jeongbin Park

**Affiliations:** School of Biomedical Convergence Engineering, Pusan National University, Yangsan, 50612 Republic of Korea

**Correction: Genom Inform 23, 26 (2025)**

**https://doi.org/10.1186/s44342-025-00057-0**

Following publication of the original article [1], the author identified an error in the competing interests section.

The incorrect competing interests section reads:

**Competing interests**

The authors declare no competing interests.

The correct competing interests section reads:

**Competing interests**

Author Jeongbin Park is an Associate Editor for Genomics & Informatics. The paper was handled by another Editor and has undergone a rigorous peer review process. Author Jeongbin Park was not involved in the journal's peer review of, or decisions related to, this manuscript.

The competing interests section has been updated above and the original article [1] has been corrected.
