# Supplementary material for: Towards a transparent and reproducible AI-assisted research paper writing
Source: Genomics Inform. 2025 Dec 2;23:26. doi: 10.1186/s44342-025-00057-0 (PMC12670809; doi:10.1186/s44342-025-00057-0)
Supplement: Supplementary file 1 — Supplementary Material 1. AI-assisted Writing Transparency Report. [file 44342_2025_57_MOESM1_ESM.docx]

AI-assisted Writing Transparency Report

**Manuscript Title:** Towards a transparent and reproducible AI-assisted research paper writing **Report Generated:** Wed, 24 Sep 2025 11:53:35 GMT

# AI MODEL INFORMATION

**Model Used:** Anthropic: Claude Sonnet 4

**Provider:** openrouter

# RESEARCH FOCUS

The following research focus was defined to generate the paper outline:

인간 참여형(Human-in-the-Loop)으로 설계된 전용 도구를 사용하여 AI를 활용한 과학 논문 작성에 대한 "Brief Report" 논문을 쓸 것임. 이 논문은 AI 기반 논문 작성에 대한 최근 논쟁에 대해 언급하고, AI 활용의 윤리적 측면에 대해서 이야기하며, 문제점을 극복하기 위한 방안으로써의 웹 기반 도구를 제안하는 것임.

- 요즘 AI가 생성한 논문이 저널에 제출되는 것을 쉽게 볼 수 있다는 점을 언급하며 글을 시작할 것임. 저널 편집장으로써, AI가 생성한 것으로 보이는 논문이 많이 제출되는 것이 일상적인 상황이 되어버린 현실을 목도함. 그러나, 우리가 이러한 논문들이 제출되는 것을 우려하는 이유에 대해서, 우리가 실제로 신경쓰는 지점은 무엇인지 다시 한번 생각해볼 필요가 있음. 정말 중요한 것은 글에서 드러나는 독창성과, 보고 내용의 재현성임. 우리는 AI가 생성한 글에 AI의 환각이 쉽게 포함될 수 있다는 사실을 알고 있고, 제출된 논문이 인간의 검증을 제대로 받지 않았을 수도 있다는 점을 우려하는 것임. 일부의 경우 제출된 논문에 인용된 문서들이 실제로는 존재하지 않는 경우 등을 예시로 들 수 있음.
- 하지만 AI에 기반한 논문 작성을 완전히 피해야 하는지에 대해서는 의문을 제기함. AI를 사용하여 논문을 작성하면서도 저자의 독창성과 결과의 재현성을 반영할 수 있는 다른 방법은 없는지 다시 한번 재고해 볼 필요가 있음. 저자의 글쓰기 실력보다 AI의 영작 실력이 더 나은 경우 (지금은 아닌 경우가 많겠지만, 앞으로는 AI의 글쓰기 실력이 비약적으로 향상될 것으로 기대함), AI는 저자들이 더 나은 영어로 글을 쓸 수 있도록 도울 수 있으며, 어쩌면 인간보다 더 명확한 메시지를 전달할 수 있을지 모름. 예를 들어, 저자의 글쓰기 능력은 우리의 무의식에 영향을 미치고 있으며 이는 논문 억셉 여부를 결정하는 데 분명 기여함. 또한, 이것이 영어가 모국어가 아닌 사람들에게 얼마나 큰 장애물이었는지, 그리고 AI가 이러한 잠재적 차별을 극복하는 데 유용할 수 있는지에 대해서 생각해 볼 필요가 있음.
- 따라서 요즘 많은 저널들은 AI를 완전히 피할 것을 요구하지 않음. 이보다는, 저자에게 AI 지원 활동에 사용되는 프롬프트(prompt)를 모두 공개해 달라고 요청하는 것이 매우 흔함. 이는 물론 앞서 이야기한 독창성과 재현성을 위한 것일 것임. 그러나, AI 프롬프트의 길이는 점점 더 길어지고 복잡해지고 있고, 따라서 이는 많은 경우 불가능한 일이 되어가고 있음. 그렇다면 과학 논문을 작성할 때 반드시 공개되어야 하는 점은 무엇인가?
- 본 저자는 이에 대한 답을 우리가 배운 글쓰기 방법에서 찾음. 우리는 과학적 글쓰기를 배울 때, 먼저 많은 논문을 읽고, 논문에 대한 전체 아이디어를 떠올린 뒤, 이를 기반으로 전체 논문의 아웃라인, 즉 각 단락별로 설명해야 할 키 포인트를 작성하고, 이렇게 작성된 아웃라인을 기반으로 글을 쓰는 방법 등을 배웠음. 이 과정에서 중요한 부분은 논문 전체에 대한 저자의 "관점"과 각 단락별 "키 포인트" 임. 따라서 논문과 함께 공개되어야 하는 점은 바로 핵심 아이디어가 요약된 이 두 가지라고 주장함.
- 따라서 본 저자는 이러한 투명성을 확보하면서도 AI를 기반으로 연구 논문을 작성하기 위한 사용하기 웹 기반 도구를 개발하였음. 이 도구는 AI를 사용하여 논문을 작성하면서도 저자의 아이디어를 논문에 그대로 담을 수 있도록 의도적으로 경직된 "단계별 구조"를 따르도록 세심하게 설계됨. 첫째, 저자가 지금까지 공부한 관련 참고문헌을 선택하는 것으로 시작함. 둘째, 선택된 참고문헌을 기반으로 저자가 AI의 도움을 받아 전체 논문 작성에 대한 명확한 관점을 정립할 수 있도록 함. 이 때, 저자는 모국어를 사용하여 관점을 작성할 수 있음. 셋째, 저자는 AI의 도움을 받아 전체 논문의 아웃라인을 정의하고 각 단락 작성의 키 포인트를 작성함. 마찬가지로, 저자는 모국어로 아웃라인과 키 포인트를 작성할 수 있음. 마지막으로, 이렇게 정리된 관점과 키 포인트를 기반으로 영문으로 된 논문을 AI가 자동으로 작성함. 이러한 각 단계를 따라, 저자는 각 단계의 결과를 검토하고 원래 저자가 생각했던 아이디어가 아닌 경우 개입하여 수정사항을 반영할 수 있도록 설계됨.
- 또한 논문이 작성된 후, "관점" 과 "키 포인트" 를 담은 "투명성 보고서" 가 제공됨. 기본적으로 이 도구는 이 두 가지 구성 요소를 기반으로 미리 정의된 AI 프롬프트를 사용하여 단락을 작성함. AI 프롬프트는 오픈 소스로 제공되므로, "관점" 과 "키 포인트" 만 공개해도 논문 작성에 사용된 완전한 프롬프트가 되며, 이 보고서 자체로도 논문의 핵심 아이디어를 쉽게 검토할 수 있도록 도움. 따라서 편집장과 리뷰어가 신속한 결정을 내리는 데 도움이 될 수 있음.
- 이 도구는 https://research.pnucolab.com에서 무료로 이용할 수 있음.
- "Acknowledgments" 섹션을 추가하고, 이 논문은 이 논문에 설명된 AI 기반 연구논문 작성 웹 도구를 사용하여 작성되었음을 명시해야 함.

다음 포맷에 맞도록 논문을 구성할 것:

Brief Report

Criteria Brief reports are suitable for the presentation of research that extends previously published research, including the reporting of additional controls and confirmatory results in other settings, as well as negative results, small-scale clinical studies, clinical audits and case series. Authors must clearly acknowledge any work upon which they are building, both published and unpublished.

Preparing your manuscript The information below details the section headings that you should include in your manuscript and what information should be within each section.

Please note that your manuscript must include a 'Declarations' section including all of the subheadings (please see below for more information).

Title page

The title page should:

present a title that includes, if appropriate, the study design e.g.: "A versus B in the treatment of C: a randomized controlled trial", "X is a risk factor for Y: a case control study", "What is the impact of factor X on subject Y: A systematic review" or for non-clinical or non-research studies: a description of what the article reports list the full names and institutional addresses for all authors if a collaboration group should be listed as an author, please list the group name as an author. If you would like the names of the individual members of the group to be searchable through their individual PubMed records, please include this information in the “Acknowledgements” section in accordance with the instructions below Large Language Models (LLMs), such as ChatGPT, do not currently satisfy our authorship criteria. Notably an attribution of authorship carries with it accountability for the work, which cannot be effectively applied to LLMs. Use of an LLM should be properly documented in the Methods section (and if a Methods section is not available, in a suitable alternative part) of the manuscript indicate the corresponding author Abstract Please minimize the use of abbreviations and do not cite references in the abstract. The abstract should briefly summarize the aim, findings or purpose of the article. The Abstract should not exceed 250 words.

Keywords Three to ten keywords representing the main content of the article.

Main text This should contain the body of the article, and may also be broken into subsections with short, informative headings.

List of abbreviations If abbreviations are used in the text they should be defined in the text at first use, and a list of abbreviations should be provided.

# PAPER OUTLINE

## 1. Abstract

- 인공지능 기반 과학 논문 작성이 학술계에서 일상적인 현실이 되었으나, AI 환각과 부정확한 인용 등의 문제로 인해 논문의 독창성과 재현성에 대한 우려가 증가하고 있음
- AI 논문 작성의 완전한 금지보다는 저자의 핵심 아이디어와 투명성을 보장하면서 AI의 언어적 장점을 활용할 수 있는 균형잡힌 접근법이 필요함
- 과학적 글쓰기에서 가장 중요한 요소는 저자의 '관점'과 각 단락별 '키 포인트'이며, 이는 AI 지원 논문 작성에서도 투명하게 공개되어야 할 핵심 정보임
- Human-in-the-Loop 방식으로 설계된 웹 기반 도구를 통해 저자가 모국어로 관점과 키 포인트를 작성하고, AI가 이를 바탕으로 영문 논문을 생성하는 단계별 구조화된 접근법을 제안함
- 투명성 보고서를 통해 저자의 핵심 아이디어를 공개함으로써 편집장과 리뷰어가 논문의 독창성을 신속하게 평가할 수 있는 새로운 검증 체계를 제시함

## 2. Introduction

- AI 생성 논문이 학술지에 제출되는 것이 일상적인 현실이 되었으며, 편집장들이 이러한 논문들을 정기적으로 접하게 됨
- AI가 비영어권 연구자들의 언어 장벽을 해결하고 과학적 소통의 형평성을 개선할 수 있는 잠재력
- AI 생성 논문의 주요 우려사항은 AI 환각현상으로 인한 부정확한 정보와 실제 존재하지 않는 허위 참고문헌 포함
- 그러나 AI 환각과 가짜 인용문헌 등의 문제로 인한 인간 검증 부재가 주요 우려사항임
- AI 생성 논문의 저널 제출이 일상화된 현실에서 우리가 진정 우려하는 핵심은 독창성과 재현성임.
- 과학적 글쓰기 교육에서 배운 핵심 요소인 '관점'과 '키 포인트'가 공개되어야 할 진정한 투명성 기준일 수 있음.
- 기존 저널들이 요구하는 AI 프롬프트 전체 공개는 프롬프트의 복잡성 증가로 인해 실용적이지 않음
- AI 도구의 완전한 금지보다는 과학적 글쓰기에서 윤리적이고 투명한 활용 방안 모색이 필요함
- Human-in-the-Loop 방식으로 설계된 웹 기반 도구를 통해 저자가 모국어로 관점과 키 포인트를 작성하고, AI가 이를 바탕으로 영문 논문을 생성하는 단계별 구조화된 접근법을 제안함

## 3. Results

- 단계별 구조를 따르는 웹 기반 도구 개발: 참고문헌 선택, 관점 정립, 아웃라인 작성, 자동 논문 생성 순서
- 저자가 모국어로 관점과 키 포인트를 작성할 수 있어 비영어권 연구자들의 언어 장벽 해결에 기여
- 각 단계에서 저자의 검토와 수정이 가능한 Human-in-the-Loop 설계로 저자의 독창성 보장
- '투명성 보고서' 제공으로 관점과 키 포인트를 공개하여 편집장과 리뷰어의 신속한 검토 지원
- 오픈 소스 AI 프롬프트와 관점, 키 포인트 공개만으로 완전한 재현성 확보
- 서버 없이 웹 브라우저 상에서 동작하는 솔루션으로 보안 강화 - LLM 또한 로컬 LLM을 고를 수 있도록 설계함
- Docker로 래핑되어 어떤 시스템에서도 쉽게 deploy 가능
- 오픈소스로 공개되어 투명성 보고서의 내용과 오픈소스 프롬프트를 조합하면 논문 작성에 활용된 완전한 프롬프트를 알 수 있음

## 4. Discussion

- AI 지원 논문 작성에서 저자성과 학술적 책임의 균형점 찾기 - 도구 사용과 인간 감독의 적절한 경계선
- 제안된 도구의 실제 구현과 무료 접근성이 학술 커뮤니티에 미칠 수 있는 긍정적 영향 전망
- https://research.pnucolab.com에서 무료 제공되는 웹 기반 도구이며 소스코드는 https://github.com/pnucolab/paper-writing-assistant 에 있음

## 5. Methods

- 이 논문은 연구에서 설명된 AI 기반 연구논문 작성 웹 도구를 사용하여 작성되었음을 명시
- AI가 자주 사용되지 않는 영어단어와 읽기 힘든 영어를 구사하는 것을 극복하기 위해 저작권에서 상대적으로 자유로운 저자의 박사 졸업 논문을 사용하여 논문을 리비전하도록 프롬프트를 작성함
