# Supplementary material for: Towards a transparent and reproducible AI-assisted research paper writing
Source: Genomics Inform. 2025 Dec 2;23:26. doi: 10.1186/s44342-025-00057-0 (PMC12670809; doi:10.1186/s44342-025-00057-0)
Supplement: Supplementary file 2 — Supplementary Material 2. Example output comparison with other tools. [file 44342_2025_57_MOESM2_ESM.docx]

**Supplementary material of**

Towards a transparent and reproducible AI-assisted research paper writing

Jeongbin Park

School of Biomedical Convergence Engineering, Pusan National University,

Yangsan, 50612, Republic of Korea

[jeongbin.park@pusan.ac.kr](mailto:jeongbin.park@pusan.ac.kr)

**Example output comparison with other tools**

Below shows three cases of generating the introduction section of an example review paper. Cases 1 and 2 are results of commercial tools, and Case 3 is that of the proposed tool. All the information provided to the tools is described. As the commercial tools do not have a function to provide citations before text generation, only prompts were given to the tools.

**Case 1**

**Tool:** Paperpal

**Prompt:** Write an introduction section on 'Cancer Genomics in the Era of Big Data: A Review’, targeting 400 words.

**Generated Text:**

| Cancer genomics has emerged as a pivotal field in oncology research, revolutionizing our understanding of cancer biology and paving the way for personalized medicine. The advent of high-throughput sequencing technologies and the exponential growth of genomic data have ushered in the era of big data in cancer research. This paradigm shift has enabled researchers to delve deeper into the molecular intricacies of cancer, uncovering novel genetic alterations, identifying potential therapeutic targets, and developing more effective treatment strategies.  The human genome project, completed in 2003, laid the foundation for large-scale genomic studies. Since then, advancements in sequencing technologies have dramatically reduced the cost and time required for whole-genome sequencing, making it feasible to analyze the genomes of thousands of tumors. This has led to the establishment of large-scale cancer genomics initiatives, such as The Cancer Genome Atlas (TCGA) and the International Cancer Genome Consortium (ICGC), which have generated vast amounts of genomic, transcriptomic, and epigenomic data from diverse cancer types.  The integration of big data analytics and machine learning algorithms with cancer genomics has opened up new avenues for data interpretation and knowledge discovery. These computational approaches enable researchers to identify complex patterns, predict cancer outcomes, and stratify patients based on their genomic profiles. Moreover, the ability to analyze large datasets has revealed the heterogeneity of cancer at both inter- and intra-tumor levels, challenging the traditional "one-size-fits-all" approach to cancer treatment.  The impact of big data in cancer genomics extends beyond basic research into clinical applications. Precision oncology, which aims to tailor treatment strategies based on individual genomic profiles, has become increasingly feasible. Genomic profiling of tumors can now guide treatment decisions, identify potential drug targets, and predict treatment responses. This approach has shown particular promise in targeted therapies and immunotherapies, where specific genetic alterations can inform the selection of appropriate interventions.  However, the era of big data in cancer genomics also presents significant challenges. The sheer volume and complexity of genomic data require sophisticated computational infrastructure and expertise for storage, processing, and analysis. Ensuring data quality, standardization, and interoperability across different platforms and studies remains a critical issue. Additionally, the interpretation of genomic data in a clinically meaningful way and the translation of research findings into actionable clinical decisions pose ongoing challenges.  As we continue to navigate the big data landscape in cancer genomics, interdisciplinary collaboration between biologists, clinicians, bioinformaticians, and data scientists becomes increasingly crucial. The integration of diverse data types, including genomics, proteomics, metabolomics, and clinical data, promises to provide a more comprehensive understanding of cancer biology and improve patient outcomes. This review aims to explore the current state of cancer genomics in the era of big data, highlighting key advancements, challenges, and future directions in this rapidly evolving field. |
| --- |

**Case 2**

**Tool:** SCISPACE

**Prompt:** Write an introduction section on 'Cancer Genomics in the Era of Big Data: A Review’, targeting 400 words.

**Generated Text:**

| The advent of big data has revolutionized many fields, and cancer genomics is no exception. As the complexity of cancer biology becomes increasingly apparent, the integration of genomic data with advanced computational tools has opened new avenues for understanding the molecular underpinnings of cancer. Cancer genomics, the study of the genetic alterations associated with cancer, has gained immense significance in recent years, driven by advancements in sequencing technologies and the increasing availability of large-scale genomic datasets. This review aims to explore the intersection of cancer genomics and big data, highlighting the transformative impact of these developments on cancer research and clinical practice.  Historically, cancer research has relied on traditional methods that often provided a limited understanding of the disease's heterogeneity. However, the emergence of next-generation sequencing (NGS) has enabled researchers to analyze the entire genome, transcriptome, and epigenome of tumor samples with unprecedented resolution. This wealth of genomic information has facilitated the identification of novel biomarkers, therapeutic targets, and insights into cancer progression and metastasis. Yet, the sheer volume of data generated poses significant challenges in terms of storage, management, and analysis.  Big data analytics has risen to meet these challenges, providing powerful tools to process and interpret complex genomic datasets. Machine learning and artificial intelligence techniques are increasingly being employed to uncover patterns and correlations within the data that would be impossible to detect through conventional analysis. These approaches not only enhance our understanding of cancer biology but also pave the way for personalized medicine, where treatments can be tailored to the specific genetic makeup of an individual's tumor.  Furthermore, the collaborative nature of big data initiatives, such as The Cancer Genome Atlas (TCGA) and Genomic Data Commons (GDC), has fostered a culture of data sharing among researchers. This collaborative spirit accelerates discoveries and allows for the validation of findings across diverse populations and cancer types, ultimately contributing to a more comprehensive understanding of cancer genomics.  In conclusion, the integration of cancer genomics with big data analytics represents a paradigm shift in the fight against cancer. As we continue to harness the power of big data, we stand on the brink of unprecedented advancements in cancer research and treatment, with the potential to significantly improve patient outcomes and revolutionize the field of oncology. This review will delve deeper into the implications of these advancements, examining current trends, challenges, and future directions in cancer genomics in the era of big data. |
| --- |

**Case 3 (proposed tool)**

**Title:** Cancer Genomics in the Era of Big Data: A Review

**Key points:**

- The advancement of high-throughput omics technologies in cancer research is generating large-scale cancer omics data, which is providing new opportunities for precision oncology (Hwang et al. 2024; Yang et al. 2025)
- Artificial intelligence, particularly deep learning technologies, is showing innovative achievements in cancer diagnosis, prognosis prediction, and treatment response prediction, transforming traditional analytical methods (Fan et al. 2024; Kather et al. 2024)
- The application of deep learning in histopathological image analysis is demonstrating significant potential in predicting molecular changes, biomarker discovery, and clinical outcome prediction (El Nahhas et al. 2025; Jiang et al. 2024)
- Multimodal deep learning approaches are playing a crucial role in understanding the complex biological mechanisms of cancer and developing personalized treatment strategies by integrating diverse omics data (Shao et al. 2024; Yuan et al. 2024)
- Challenges such as model interpretability, clinical validation, and integration with existing medical workflows still exist in the clinical application of AI-based cancer analysis (Ramon et al. 2024; Unger & Kather 2024)

**Citations:**

[1] "A thousand and one tumors: the promise of AI for cancer biology" by Joy Linyue Fan, Achille Nazaret & Elham Azizi (2024)

Abstract: Breakthroughs in AI and multimodal genomics are unlocking the ability to study the tumor microenvironment. We explore promising machine learning techniques to integrate and interpret high-dimensional data, examine cellular dynamics and unravel gene regulatory mechanisms, ultimately enhancing our understanding of tumor progression and resistance.

[2] "Big data and deep learning for RNA biology" by Hyeonseo Hwang, Hyeonseong Jeon, Nagyeong Yeo, Daehyun Baek (2024)

Abstract: The exponential growth of big data in RNA biology (RB) has led to the development of deep learning (DL) models that have driven crucial discoveries. As constantly evidenced by DL studies in other fields, the successful implementation of DL in RB depends heavily on the effective utilization of large-scale datasets from public databases. In achieving this goal, data encoding methods, learning algorithms, and techniques that align well with biological domain knowledge have played pivotal roles. In this review, we provide guiding principles for applying these DL concepts to various problems in RB by demonstrating successful examples and associated methodologies. We also discuss the remaining challenges in developing DL models for RB and suggest strategies to overcome these challenges. Overall, this review aims to illuminate the compelling potential of DL for RB and ways to apply this powerful technology to investigate the intriguing biology of RNA more effectively.

[3] "Enhancing Lung Cancer Classification and Prediction With Deep Learning and Multi-Omics Data" by Tehnan I. A. Mohamed, Absalom El-Shamir Ezugwu (2024)

Abstract: Lung adenocarcinoma (LUAD), a prevalent histological type of lung cancer and a subtype of non-small cell lung cancer (NSCLC) accounts for 45–55% of all lung cancer cases. Various factors, including environmental influences and genetics, have been identified as contributors to the initiation and progression of LUAD. Recent large-scale analyses have probed into RNASeq, miRNA, and DNA methylation alterations in LUAD. In this study, we devised an innovative deep-learning model for lung cancer detection by integrating markers from mRNA, miRNA, and DNA methylation. The initial phase involved meticulous data preparation, encompassing multiple steps, followed by a differential analysis aimed at identifying genes exhibiting differential expression across different lung cancer stages (Stages I, II, III, and IV). The DESeq2 technique was employed for RNASeq data, while the LIMMA package was utilized for miRNA and DNA methylation datasets during the differential analysis. Subsequently, integration of all prepared omics data types was achieved by selecting common samples, resulting in a consolidated dataset comprising 448 samples and 8228 features (genes). To streamline features, principal components analysis (PCA) was implemented, and the synthetic minority over-sampling technique (SMOTE) algorithm was applied to ensure class balance. The integrated and processed data were then input into the PCA-SMOTE-CNN model for the classification process. The deep learning model, specifically designed for classifying and predicting lung cancer using an integrated omics dataset, was evaluated using various metrics, including precision, recall, F1-score, and accuracy. Experimental results emphasized the superior predictive performance of the proposed model, attaining an accuracy, precision, recall, and F1-score of 0.97 each, surpassing recent competitive methods.

[4] "From whole-slide image to biomarker prediction: end-to-end weakly supervised deep learning in computational pathology" by Omar S. M. El Nahhas, Marko van Treeck, Georg Wölflein, Michaela Unger, Marta Ligero, Tim Lenz, Sophia J. Wagner, Katherine J. Hewitt, Firas Khader, Sebastian Foersch, Daniel Truhn, Jakob Nikolas Kather (2025)

Abstract: Hematoxylin- and eosin-stained whole-slide images (WSIs) are the foundation of diagnosis of cancer. In recent years, development of deep learning-based methods in computational pathology has enabled the prediction of biomarkers directly from WSIs. However, accurately linking tissue phenotype to biomarkers at scale remains a crucial challenge for democratizing complex biomarkers in precision oncology. This protocol describes a practical workflow for solid tumor associative modeling in pathology (STAMP), enabling prediction of biomarkers directly from WSIs by using deep learning. The STAMP workflow is biomarker agnostic and allows for genetic and clinicopathologic tabular data to be included as an additional input, together with histopathology images. The protocol consists of five main stages that have been successfully applied to various research problems: formal problem definition, data preprocessing, modeling, evaluation and clinical translation. The STAMP workflow differentiates itself through its focus on serving as a collaborative framework that can be used by clinicians and engineers alike for setting up research projects in the field of computational pathology. As an example task, we applied STAMP to the prediction of microsatellite instability (MSI) status in colorectal cancer, showing accurate performance for the identification of tumors high in MSI. Moreover, we provide an open-source code base, which has been deployed at several hospitals across the globe to set up computational pathology workflows. The STAMP workflow requires one workday of hands-on computational execution and basic command line knowledge.

[5] "Deep learning for dual detection of microsatellite instability and POLE mutations in colorectal cancer histopathology" by Marco Gustav, Nic Gabriel Reitsam, Zunamys I. Carrero, Chiara M. L. Loeffler, Marko van Treeck, Tanwei Yuan, Nicholas P. West, Philip Quirke, Titus J. Brinker, Hermann Brenner, Loëtitia Favre, Bruno Märkl, Albrecht Stenzinger, Alexander Brobeil, Michael Hoffmeister, Julien Calderaro, Anaïs Pujals, Jakob Nikolas Kather (2024)

Abstract: In the spectrum of colorectal tumors, microsatellite-stable (MSS) tumors with DNA polymerase ε (POLE) mutations exhibit a hypermutated profile, holding the potential to respond to immunotherapy similarly to their microsatellite-instable (MSI) counterparts. Yet, due to their rarity and the associated testing costs, systematic screening for these mutations is not commonly pursued. Notably, the histopathological phenotype resulting from POLE mutations is theorized to resemble that of MSI. This resemblance not only could facilitate their detection by a transformer-based Deep Learning (DL) system trained on MSI pathology slides, but also indicates the possibility for MSS patients with POLE mutations to access enhanced treatment options, which might otherwise be overlooked. To harness this potential, we trained a Deep Learning classifier on a large dataset with the ground truth for microsatellite status and subsequently validated its capabilities for MSI and POLE detection across three external cohorts. Our model accurately identified MSI status in both the internal and external resection cohorts using pathology images alone. Notably, with a classification threshold of 0.5, over 75% of POLE driver mutant patients in the external resection cohorts were flagged as "positive" by a DL system trained on MSI status. In a clinical setting, deploying this DL model as a preliminary screening tool could facilitate the efficient identification of clinically relevant MSI and POLE mutations in colorectal tumors, in one go.

[6] "Deep learning applications in breast cancer histopathological imaging: diagnosis, treatment, and prognosis" by Bitao Jiang, Lingling Bao, Songqin He, Xiao Chen, Zhihui Jin, Yingquan Ye (2024)

Abstract: Breast cancer is the most common malignant tumor among women worldwide and remains one of the leading causes of death among women. Its incidence and mortality rates are continuously rising. In recent years, with the rapid advancement of deep learning (DL) technology, DL has demonstrated significant potential in breast cancer diagnosis, prognosis evaluation, and treatment response prediction. This paper reviews relevant research progress and applies DL models to image enhancement, segmentation, and classification based on large-scale datasets from TCGA and multiple centers. We employed foundational models such as ResNet50, Transformer, and Hover-net to investigate the performance of DL models in breast cancer diagnosis, treatment, and prognosis prediction. The results indicate that DL techniques have significantly improved diagnostic accuracy and efficiency, particularly in predicting breast cancer metastasis and clinical prognosis. Furthermore, the study emphasizes the crucial role of robust databases in developing highly generalizable models. Future research will focus on addressing challenges related to data management, model interpretability, and regulatory compliance, ultimately aiming to provide more precise clinical treatment and prognostic evaluation programs for breast cancer patients.

[7] "Deep Learning Model for Predicting Immunotherapy Response in Advanced Non-Small Cell Lung Cancer" by Mehrdad Rakaee, PhD; Masoud Tafavvoghi, MSc; Biagio Ricciuti, MD; Joao V. Alessi, MD; Alessio Cortellini, MD, PhD; Fabrizio Citarella, MD; Lorenzo Nibid, MD; Giuseppe Perrone, MD; Elio Adib, MD; Claudia A. M. Fulgenzi, MD; Cassio Murilo Hidalgo Filho, MD; Alessandro Di Federico, MD; Falah Jabar, PhD; Sayed Hashemi, MD; Ilias Houda, MD; Elin Richardsen, MD, PhD; Lill-Tove Rasmussen Busund, MD, PhD; Tom Donnem, MD, PhD; Idris Bahce, MD, PhD; David J. Pinato, MD, PhD; Åslaug Helland, MD, PhD; Lynette M. Sholl, MD; Mark M. Awad, MD, PhD; David J. Kwiatkowski, MD, PhD (2025)

Abstract: IMPORTANCE Only a small fraction of patients with advanced non−small cell lung cancer (NSCLC) respond to immune checkpoint inhibitor (ICI) treatment. For optimal personalized NSCLC care, it is imperative to identify patients who are most likely to benefit from immunotherapy. OBJECTIVE To develop a supervised deep learning−based ICI response prediction method; evaluate its performance alongside other known predictive biomarkers; and assess its association with clinical outcomes in patients with advanced NSCLC. DESIGN, SETTING, AND PARTICIPANTS This multicenter cohort study developed and independently validated a deep learning−based response stratification model for predicting ICI treatment outcome in patients with advanced NSCLC from whole slide hematoxylin and eosin–stained images. Images for model development and validation were obtained from 1 participating center in the US and 3 in the European Union (EU) from August 2014 to December 2022. Data analyses were performed from September 2022 to May 2024. EXPOSURE Monotherapy with ICIs. MAIN OUTCOMES AND MEASURES Model performance measured by clinical end points and objective response rate (ORR) differentiation power vs other predictive biomarkers, ie, programmed death-ligand 1 (PD-L1), tumor mutational burden (TMB), and tumor-infiltrating lymphocytes (TILs). RESULTS A total of 295 581 image tiles from 958 patients (mean [SD] age, 66.0 [10.6] years; 456 [48%] females and 502 [52%] males) treated with ICI for NSCLC were included in the analysis. The US-based development cohort consisted of 614 patients with median (IQR) follow-up time of 54.5 (38.2-68.1) months, and the EU-based validation cohort, 344 patients with 43.3 (27.4-53.9) months of follow-up. The ORR to ICI was 26% in the developmental cohort and 28% in the validation cohort. The deep learning model's area under the receiver operating characteristic curve (AUC) for ORR was 0.75 (95% CI, 0.64-0.85) in the internal test set and 0.66 (95% CI, 0.60-0.72) in the validation cohort. In a multivariable analysis, the deep learning model's score was an independent predictor of ICI response in the validation cohort for both progression-free (hazard ratio, 0.56; 95% CI, 0.42-0.76;P < .001) and overall survival (hazard ratio, 0.53; 95% CI, 0.39-0.73;P < .001). The tuned deep learning model achieved a higher AUC than TMB, TILs, and PD-L1 in the internal set; in the validation cohort, it was superior to TILs and comparable with PD-L1 (AUC, 0.67; 95% CI, 0.60-0.74), with a 10-percentage point improvement in specificity. In the validation cohort, combining the deep learning model with PD-L1 scores achieved an AUC of 0.70 (95% CI, 0.63-0.76), outperforming either marker alone, with a response rate of 51% compared to 41% for PD-L1 alone. CONCLUSIONS AND RELEVANCE The findings of this cohort study demonstrate a strong and independent deep learning−based feature associated with ICI response in patients with NSCLC across various cohorts. Clinical use of this deep learning model could refine treatment precision and better identify patients who are likely to benefit from ICI for treatment of advanced NSCLC.

[8] "Multi-Instance Multi-Task Learning for Joint Clinical Outcome and Genomic Profile Predictions From the Histopathological Images" by Wei Shao, Hang Shi, Jianxin Liu, Yingli Zuo, Liang Sun, Tiansong Xia, Wanyuan Chen, Peng Wan, Jianpeng Sheng, Qi Zhu,, Daoqiang Zhang (2024)

Abstract: With the remarkable success of digital histopathology and the deep learning technology, many whole-slide pathological images (WSIs) based deep learning models are designed to help pathologists diagnose human cancers. Recently, rather than predicting categorical variables as in cancer diagnosis, several deep learning studies are also proposed to estimate the continuous variables such as the patients' survival or their transcriptional profile. However, most of the existing studies focus on conducting these predicting tasks separately, which overlooks the useful intrinsic correlation among them that can boost the prediction performance of each individual task. In addition, it is sill challenge to design the WSI-based deep learning models, since a WSI is with huge size but annotated with coarse label. In this study, we propose a general multi-instance multi-task learning framework (HistMIMT) for multi-purpose prediction from WSIs. Specifically, we firstly propose a novel multi-instance learning module (TMICS) considering both common and specific task information across different tasks to generate bag representation for each individual task. Then, a soft-mask based fusion module with channel attention (SFCA) is developed to leverage useful information from the related tasks to help improve the prediction performance on target task. We evaluate our method on three cancer cohorts derived from the Cancer Genome Atlas (TCGA). For each cohort, our multi-purpose prediction tasks range from cancer diagnosis, survival prediction and estimating the transcriptional profile of gene TP53. The experimental results demonstrated that HistMIMT can yield better outcome on all clinical prediction tasks than its competitors.

[9] "Multimodal data integration using deep learning predicts overall survival of patients with glioma" by Yifan Yuan, Xuan Zhang, Yining Wang, Hongyan Li, Zengxin Qi, Zunguo Du, Ying-Hua Chu, Danyang Feng, Jie Hu, Qingguo Xie, Jianping Song, Yuqing Liu, Jiajun Cai (2024)

Abstract: Gliomas are highly heterogeneous diseases with poor prognosis. Precise survival prediction could benefit further clinical decision-making, clinical trial incursion, and health economics. Recent research has emphasized the prognostic value of magnetic resonance imaging, pathological specimens, and circulating biomarkers. However, the integrative potential and efficacy of these modalities require to be further validated. After incorporating 218 patients of The Cancer Genome Atlas glioma datasets of and 54 patients of the Huashan cohort with complementary prognostic information, we established a squeeze-and-excitation deep learning feature extractor for T1-contrast enhanced images and histological slides and explored to screen significant circulating 5-hydroxymethylcytosines (5hmC) profiles for glioma survival by least absolute shrinkage and selection operator-Cox regression. Therefore, a prognostication predictive model with high efficiency was obtained through survival support vector machine multimodal integration of radiologic imaging, histopathologic imaging features, [abstract appears to be cut off]

[10] "Development and deployment of a histopathology-based deep learning algorithm for patient prescreening in a clinical trial" by Albert Juan Ramon, Chaitanya Parmar, Oscar M. Carrasco-Zevallos, Carlos Csiszer, Stephen S. F. Yip, Patricia Raciti, Nicole L. Stone, Spyros Triantos, Michelle M. Quiroz, Patrick Crowley, Ashita S. Batavia, Joel Greshock, Tommaso Mansi, Kristopher A. Standish (2024)

Abstract: Accurate identification of genetic alterations in tumors, such as Fibroblast Growth Factor Receptor, is crucial for treating with targeted therapies; however, molecular testing can delay patient care due to the time and tissue required. Successful development, validation, and deployment of an AI-based, biomarker-detection algorithm could reduce screening cost and accelerate patient recruitment. Here, we develop a deep-learning algorithm using >3000 H&E-stained whole slide images from patients with advanced urothelial cancers, optimized for high sensitivity to avoid ruling out trial-eligible patients. The algorithm is validated on a dataset of 350 patients, achieving an area under the curve of 0.75, specificity of 31.8% at 88.7% sensitivity, and projected 28.7% reduction in molecular testing. We successfully deploy the system in a non-interventional study comprising 89 global study clinical sites and demonstrate its potential to prioritize/deprioritize molecular testing resources and provide substantial cost savings in the drug development and clinical settings.

[11] "Omics-based deep learning approaches for lung cancer decision-making and therapeutics development" by Thi-Oanh Tran, Thanh Hoa Vo, Nguyen Quoc Khanh Le (2024)

Abstract: Lung cancer has been the most common and the leading cause of cancer deaths globally. Besides clinicopathological observations and traditional molecular tests, the advent of robust and scalable techniques for nucleic acid analysis has revolutionized biological research and medicinal practice in lung cancer treatment. In response to the demands for minimally invasive procedures and technology development over the past decade, many types of multi-omics data at various genome levels have been generated. As omics data grow, artificial intelligence models, particularly deep learning, are prominent in developing more rapid and effective methods to potentially improve lung cancer patient diagnosis, prognosis and treatment strategy. This decade has seen genome-based deep learning models thriving in various lung cancer tasks, including cancer prediction, subtype classification, prognosis estimation, cancer molecular signatures identification, treatment response prediction and biomarker development. In this study, we summarized available data sources for deep-learning-based lung cancer mining and provided an update on recent deep learning models in lung cancer genomics. Subsequently, we reviewed the current issues and discussed future research directions of deep-learning-based lung cancer genomics research.

[12] "Biologically interpretable multi-task deep learning pipeline predicts molecular alterations, grade, and prognosis in glioma patients" by Xuewei Wu, Shuaitong Zhang, Zhenyu Zhang, Zicong He, Zexin Xu, Weiwei Wang, Zhe Jin, Jingjing You, Yang Guo, Lu Zhang, Wenhui Huang, Fei Wang, Xianzhi Liu, Dongming Yan, Jingliang Cheng, Jing Yan, Shuixing Zhang, Bin Zhang (2024)

Abstract: Deep learning models have been developed for various predictions in glioma; yet, they were constrained by manual segmentation, task-specific design, or a lack of biological interpretation. Herein, we aimed to develop an end-to-end multi-task deep learning (MDL) pipeline that can simultaneously predict molecular alterations and histological grade (auxiliary tasks), as well as prognosis (primary task) in gliomas. Further, we aimed to provide the biological mechanisms underlying the model's predictions. We collected multiscale data including baseline MRI images from 2776 glioma patients across two private (FAHZU and HPPH, n = 1931) and three public datasets (TCGA, n = 213; UCSF, n = 410; and EGD, n = 222). We trained and internally validated the MDL model using our private datasets, and externally validated it using the three public datasets. We used the model-predicted deep prognosis score (DPS) to stratify patients into low-DPS and high-DPS subtypes. Additionally, a radio-multiomics analysis was conducted to elucidate the biological basis of the DPS. In the external validation cohorts, the MDL model achieved average areas under the curve of 0.892–0.903, 0.710–0.894, and 0.850–0.879 for predicting IDH mutation status, 1p/19q co-deletion status, and tumor grade, respectively. Moreover, the MDL model yielded a C-index of 0.723 in the TCGA and 0.671 in the UCSF for the prediction of overall survival. The DPS exhibits significant correlations with activated oncogenic pathways, immune infiltration patterns, specific protein expression, DNA methylation, tumor mutation burden, and tumor-stroma ratio. Accordingly, our work presents an accurate and biologically meaningful tool for predicting molecular subtypes, tumor grade, and survival outcomes in gliomas, which provides personalized clinical decision-making in a global and non-invasive manner.

[13] "End-to-end prognostication in colorectal cancer by deep learning: a retrospective, multicentre study" by Xiaofeng Jiang, Michael Hoffmeister, Hermann Brenner, Hannah Sophie Muti, Tanwei Yuan, Sebastian Foersch, Nicholas P West, Alexander Brobeil, Jitendra Jonnagaddala, Nicholas Hawkins, Robyn L Ward, Titus J Brinker, Oliver Lester Saldanha, Jia Ke, Wolfram Müller, Heike I Grabsch, Philip Quirke, Daniel Truhn, Jakob Nikolas Kather (2024)

Abstract: Background Precise prognosis prediction in patients with colorectal cancer (ie, forecasting survival) is pivotal for individualised treatment and care. Histopathological tissue slides of colorectal cancer specimens contain rich prognostically relevant information. However, existing studies do not have multicentre external validation with real-world sample processing protocols, and algorithms are not yet widely used in clinical routine. Methods In this retrospective, multicentre study, we collected tissue samples from four groups of patients with resected colorectal cancer from Australia, Germany, and the USA. We developed and externally validated a deep learning-based prognostic-stratification system for automatic prediction of overall and cancer-specific survival in patients with resected colorectal cancer. We used the model-predicted risk scores to stratify patients into different risk groups and compared survival outcomes between these groups. Additionally, we evaluated the prognostic value of these risk groups after adjusting for established prognostic variables. Findings We trained and validated our model on a total of 4428 patients. We found that patients could be divided into high-risk and low-risk groups on the basis of the deep learning-based risk score. On the internal test set, the group with a high-risk score had a worse prognosis than the group with a low-risk score, as reflected by a hazard ratio (HR) of 4·50 (95% CI 3·33–6·09) for overall survival and 8·35 (5·06–13·78) for disease-specific survival (DSS). We found consistent performance across three large external test sets. In a test set of 1395 patients, the high-risk group had a lower DSS than the low-risk group, with an HR of 3·08 (2·44–3·89). In two additional test sets, the HRs for DSS were 2·23 (1·23–4·04) and 3·07 (1·78–5·3). We showed that the prognostic value of the deep learning-based risk score is independent of established clinical risk factors. Interpretation Our findings indicate that attention-based self-supervised deep learning can robustly offer a prognosis on clinical outcomes in patients with colorectal cancer, generalising across different populations and serving as a potentially new prognostic tool in clinical decision making for colorectal cancer management. We release all source codes and trained models under an open-source licence, allowing other researchers to reuse and build upon our work.

[14] "A systematic analysis of deep learning in genomics and histopathology for precision oncology" by Michaela Unger, Jakob Nikolas Kather (2024)

Abstract: Background Digitized histopathological tissue slides and genomics profiling data are available for many patients with solid tumors. In the last 5 years, Deep Learning (DL) has been broadly used to extract clinically actionable information and biological knowledge from pathology slides and genomic data in cancer. In addition, a number of recent studies have introduced multimodal DL models designed to simultaneously process both images from pathology slides and genomic data as inputs. By comparing patterns from one data modality with those in another, multimodal DL models are capable of achieving higher performance compared to their unimodal counterparts. However, the application of these methodologies across various tumor entities and clinical scenarios lacks consistency. Methods Here, we present a systematic survey of the academic literature from 2010 to November 2023, aiming to quantify the application of DL for pathology, genomics, and the combined use of both data types. After filtering 3048 publications, our search identified 534 relevant articles which then were evaluated by basic (diagnosis, grading, subtyping) and advanced (mutation, drug response and survival prediction) application types, publication year and addressed cancer tissue. Results Our analysis reveals a predominant application of DL in pathology compared to genomics. However, there is a notable surge in DL incorporation within both domains. Furthermore, while DL applied to pathology primarily targets the identification of histology-specific patterns in individual tissues, DL in genomics is more commonly used in a pan-cancer context. Multimodal DL, on the contrary, remains a niche topic, evidenced by a limited number of publications, primarily focusing on prognosis predictions. Conclusion In summary, our quantitative analysis indicates that DL not only has a well-established role in histopathology but is also being successfully integrated into both genomic and multimodal applications. In addition, there is considerable potential in multimodal DL for harnessing further advanced tasks, such as predicting drug response. Nevertheless, this review also underlines the need for further research to bridge the existing gaps in these fields.

[15] "Morphological diversity of cancer cells predicts prognosis across tumor types" by Rasoul Sali, Yuming Jiang, Armin Attaranzadeh, Brittany Holmes, Ruijiang Li (2024)

Abstract: Background: Intratumor heterogeneity drives disease progression and treatment resistance, which can lead to poor patient outcomes. Here, we present a computational approach for quantification of cancer cell diversity in routine hematoxylin-eosin–stained histopathology images.

[16] "Deep learning framework for comprehensive molecular and prognostic stratifications of triple-negative breast cancer" by Shen Zhao, Chao-Yang Yan, Hong Lv, Jing-Cheng Yang, Chao You, Zi-Ang Li, Ding Ma, Yi Xiao, Jia Hu, Wen-Tao Yang, Yi-Zhou Jiang, Jun Xu, Zhi-Ming Shao (2024)

Abstract: Triple-negative breast cancer (TNBC) is the most challenging breast cancer subtype. Molecular stratification and target therapy bring clinical benefit for TNBC patients, but it is difficult to implement comprehensive molecular testing in clinical practice. Here, using our multi-omics TNBC cohort (N = 425), a deep learning-based framework was devised and validated for comprehensive predictions of molecular features, subtypes and prognosis from pathological whole slide images. The framework first incorporated a neural network to decompose the tissue on WSIs, followed by a second one which was trained based on certain tissue types for predicting different targets. Multi-omics molecular features were analyzed including somatic mutations, copy number alterations, germline mutations, biological pathway activities, metabolomics features and immunotherapy biomarkers. It was shown that the molecular features with therapeutic implications can be predicted including the somatic PIK3CA mutation, germline BRCA2 mutation and PD-L1 protein expression (area under the curve [AUC]: 0.78, 0.79 and 0.74 respectively). The molecular subtypes of TNBC can be identified (AUC: 0.84, 0.85, 0.93 and 0.73 for the basal-like immune-suppressed, immunomodulatory, luminal androgen receptor, and mesenchymal-like subtypes respectively) and their distinctive morphological patterns were revealed, which provided novel insights into the heterogeneity of TNBC. A neural network integrating image features and clinical covariates stratified patients into groups with different survival outcomes (log-rank P < 0.001). Our prediction framework and neural network models were externally validated on the TNBC cases from TCGA (N = 143) and appeared robust to the changes in patient population. For potential clinical translation, we built a novel online platform, where we modularized and deployed our framework along with the validated models. It can realize real-time one-stop prediction for new cases. In summary, using only pathological WSIs, our proposed framework can enable comprehensive stratifications of TNBC patients and provide valuable information for therapeutic decision-making. It had the potential to be clinically implemented and promote the personalized management of TNBC.

[17] "Harnessing TME depicted by histological images to improve cancer prognosis through a deep learning system" by Ruitian Gao, Xin Yuan, Yanran Ma, ..., Jing Sun, Yu Guang Wang, Zhangsheng Yu (2024)

Abstract: [The provided text contains a brief summary rather than a full abstract: "Gao et al. provide a powerful deep learning system for TME analysis and cancer prognosis from the aspect of histological images. The system can enhance survival prediction accuracy for all publicly available cancer cohorts using only histological images without the need for ST data."]

[18] "Multimodal Non-Small Cell Lung Cancer Classification Using Convolutional Neural Networks" by Marian Magdy Amin, Ahmed S. Ismail, Masoud E. Shaheen (2024)

Abstract: Lung cancer is the leading cause of death worldwide. Early detection of lung cancer is a hard mission. New Small Cell Lung Cancer (NSCLC) is the most prevalent sub-type of lung cancer. Differentiating between several NSCLC subtypes is important for making the right decision of treatment plan for the patient. Despite the focus of recent researchers on single modality approach, multi-omics modalities have many underlying influences and discoveries in the cancer detection and classification area. Through this research multi-omics modalities are used. Previous efforts have been focused either on multimodality using traditional machine learning classifiers or single modality using deep learning. Also, for the molecular sources (RNA-seq and miRNA-Seq) traditional machine learning approaches are usually used. For this work, deep learning using Convolutional Neural Networks (CNNs) is used and applied on the above-mentioned multimodalities. The classification accuracy results obtained for RNA-Seq, miRNA-Seq, WSIs are 96.79%, 98.59%, 89.73% respectively. The F1 scores obtained for RNA-Seq, miRNA-Seq, WSIs are 95.238%,99.67%,89.76% respectively. Moreover, the Area Under Curve obtained for RNA-Seq, miRNA-Seq, WSIs are 100%, 99.41%,97,54% respectively. These results improves the results obtained by other related works as will be explained. According to these improvements in the results, the lung cancer classification could be better and the disease would be discovered at early stages which is the goal for the research field efforts.

[19] "Colon cancer survival prediction from gland shapes within histology slides using deep learning" by Rawan Gedeon, Atulya Nagar (2025)

Abstract: This study investigates the application of deep learning techniques for segmenting glands in histopathological images of colorectal cancer. We trained two convolutional neural network models, U-Net and DCAN, on a combination of the GlaS and CRAG datasets to enhance generalization across diverse histological appearances, selecting DCAN for its superior accuracy in delineating gland boundaries. The goal was to achieve robust gland segmentation applicable to whole slide images (WSIs) from The Cancer Genome Atlas (TCGA). Using the segmented glands, we extracted patient-level morphological features and used them to predict survival outcomes. A Cox proportional hazards model was trained on these features and achieved a high concordance index, indicating strong predictive performance. Patients were then stratified into high- and low-risk groups, with significant differences in survival distributions (log-rank p-value: 0.01317). In addition, we benchmarked our models against state-of-the-art gland segmentation methods on GlaS and CRAG, highlighting the trade-off between domain-specific accuracy and cross-dataset robustness.

[20] "Deep learning in cancer genomics and histopathology" by Michaela Unger, Jakob Nikolas Kather (2024)

Abstract: Histopathology and genomic profiling are cornerstones of precision oncology and are routinely obtained for patients with cancer. Traditionally, histopathology slides are manually reviewed by highly trained pathologists. Genomic data, on the other hand, is evaluated by engineered computational pipelines. In both applications, the advent of modern artificial intelligence methods, specifically machine learning (ML) and deep learning (DL), have opened up a fundamentally new way of extracting actionable insights from raw data, which could augment and potentially replace some aspects of traditional evaluation workflows. In this review, we summarize current and emerging applications of DL in histopathology and genomics, including basic diagnostic as well as advanced prognostic tasks. Based on a growing body of evidence, we suggest that DL could be the groundwork for a new kind of workflow in oncology and cancer research. However, we also point out that DL models can have biases and other flaws that users in healthcare and research need to know about, and we propose ways to address them.

[21] "Mutations in Anaplastic Thyroid Carcinoma: An Analysis of the Japanese National Genomic Database" by Hiromi Nagano, Hayato Matsumoto, Yumi Ando, Masatoyo Nakajo, Masaru Yamashita (2025)

Abstract: Objectives: The purpose of this study is to investigate the genetic mutational status of anaplastic thyroid carcinoma (ATC) and its prognostic implications.

[22] "Multimodal deep learning approaches for precision oncology: a comprehensive review" by Huan Yang, Minglei Yang, Jiani Chen, Guocong Yao, Quan Zou, Linpei Jia (2025)

Abstract: The burgeoning accumulation of large-scale biomedical data in oncology, alongside significant strides in deep learning (DL) technologies, has established multimodal DL (MDL) as a cornerstone of precision oncology. This review provides an overview of MDL applications in this field, based on an extensive literature survey. In total, 651 articles published before September 2024 are included. We first outline publicly available multimodal datasets that support cancer research. Then, we discuss key DL training methods, data representation techniques, and fusion strategies for integrating multimodal data. The review also examines MDL applications in tumor segmentation, detection, diagnosis, prognosis, treatment selection, and therapy response monitoring. Finally, we critically assess the limitations of current approaches and propose directions for future research. By synthesizing current progress and identifying challenges, this review aims to guide future efforts in leveraging MDL to advance precision oncology.

[23] "Deeply integrating latent consistent representations in high-noise multi-omics data for cancer subtyping" by Yueyi Cai, Shunfang Wang (2024)

Abstract: Cancer is a complex and high-mortality disease regulated by multiple factors. Accurate cancer subtyping is crucial for formulating personalized treatment plans and improving patient survival rates. The underlying mechanisms that drive cancer progression can be comprehensively understood by analyzing multi-omics data. However, the high noise levels in omics data often pose challenges in capturing consistent representations and adequately integrating their information. This paper proposed a novel variational autoencoder-based deep learning model, named Deeply Integrating Latent Consistent Representations (DILCR). Firstly, multiple independent variational autoencoders and contrastive loss functions were designed to separate noise from omics data and capture latent consistent representations. Subsequently, an Attention Deep Integration Network was proposed to integrate consistent representations across different omics levels effectively. Additionally, we introduced the Improved Deep Embedded Clustering algorithm to make integrated variable clustering friendly. The effectiveness of DILCR was evaluated using 10 typical cancer datasets from The Cancer Genome Atlas and compared with 14 state-of-the-art integration methods. The results demonstrated that DILCR effectively captures the consistent representations in omics data and outperforms other integration methods in cancer subtyping. In the Kidney Renal Clear Cell Carcinoma case study, cancer subtypes were identified by DILCR with significant biological significance and interpretability.

[24] "Current computational methods for spatial transcriptomics in cancer biology" by Jaewoo Mo, Junseong Bae, Jahanzeb Saqib, Dohyun Hwang, Yunjung Jin, Beomsu Park, Jeongbin Park, Junil Kim (2024)

Abstract: Cells in multicellular organisms constitute a self-organizing society by interacting with their neighbors. Cancer originates from malfunction of cellular behavior in the context of such a self-organizing system. The identities or characteristics of individual tumor cells can be represented by the hallmark of gene expression or transcriptome, which can be addressed using single-cell dissociation followed by RNA sequencing. However, the dissociation process of single cells results in losing the cellular address in tissue or neighbor information of each tumor cell, which is critical to understanding the malfunctioning cellular behavior in the microenvironment. Spatial transcriptomics technology enables measuring the transcriptome which is tagged by the address within a tissue. However, to understand cellular behavior in a self-organizing society, we need to apply mathematical or statistical methods. Here, we provide a review on current computational methods for spatial transcriptomics in cancer biology.

[25] "Advances in machine learning for tumour classification in cancer of unknown primary: A mini-review" by Karen Oróstica, Felipe Mardones, Yanara A. Bernal, Samuel Molina, Marcos Orchard, Ricardo A. Verdugo, Daniel Carvajal-Hausdorf, Katherine Marcelain, Seba Contreras, Ricardo Armisen (2025)

Abstract: Cancers of unknown primary (CUP) are a heterogeneous group of aggressive metastatic cancers where standardised diagnostic techniques fail to identify the organ where it originated, resulting in a poor prognosis and resistance to treatment. Recent advances in large-scale sequencing techniques have enabled the identification of mutational signatures specific to particular tumour subtypes, even from liquid biopsy samples such as blood. This breakthrough paves the way for the development of new cost-effective diagnostic strategies. This mini-review explores recent advancements in Machine Learning (ML) and its application to tumour classification methods for CUP patients, identifying its weaknesses and strengths when classifying the tumour type. In the era of multi-omics, integrating several sources of information (e.g., imaging, molecular biomarkers, and family history) requires important theoretical advancements: increasing the dimensionality of the problem can result in lowering the predictive accuracy and robustness when data is scarce. Here, we review and discuss different architectures and strategies for incorporating cutting-edge machine learning into CUP diagnosis, aiming to bridge the gap between theory and clinical practice.

[26] "Deep Learning Artificial Intelligence Predicts Homologous Recombination Deficiency and Platinum Response From Histologic Slides" by Erik N. Bergstrom, Ammal Abbasi, Marcos Díaz-Gay, Loïck Galland, Sylvain Ladoire, Scott M. Lippman,, Ludmil B. Alexandrov (2024)

Abstract: PURPOSE Cancers with homologous recombination deficiency (HRD) can benefit from platinum salts and poly(ADP-ribose) polymerase inhibitors. Standard diagnostic tests for detecting HRD require molecular profiling, which is not universally available. METHODS We trained DeepHRD, a deep learning platform for predicting HRD from hematoxylin and eosin (H&E)–stained histopathological slides, using primary breast (n=1,008) and ovarian (n=459) cancers from The Cancer Genome Atlas (TCGA). DeepHRD was compared with four standard HRD molecular tests using breast (n=349) and ovarian (n=141) cancers from multiple independent data sets, including platinum-treated clinical cohorts with RECIST progression-free survival (PFS), complete response (CR), and overall survival (OS) endpoints. RESULTS DeepHRD predicted HRD from held-out H&E-stained breast cancer slides in TCGA with an AUC of 0.81 (95% CI, 0.77 to 0.85). This performance was confirmed in two independent primary breast cancer cohorts (AUC, 0.76 [95% CI, 0.71 to 0.82]). In an external platinum-treated metastatic breast cancer cohort, samples predicted as HRD had higher complete CR (AUC, 0.76 [95% CI, 0.54 to 0.93]) with 3.7-fold increase in median PFS (14.4v 3.9 months; P = .0019) and hazard ratio (HR) of 0.45 (P = .0047). There were no significant differences in nonplatinum treatment outcome by predicted HRD status in three breast cancer cohorts, including CR (AUC, 0.39) and PFS (HR, 0.98, P = .95) in taxane-treated metastatic breast cancer. Through transfer learning to high-grade serous ovarian cancer, DeepHRD-predicted HRD samples had better OS after first-line (HR, 0.46; P = .030) and neoadjuvant (HR, 0.49; P = .015) platinum therapy in two cohorts. CONCLUSION DeepHRD can predict HRD in breast and ovarian cancers directly from routine H&E slides across multiple external cohorts, slide scanners, and tissue fixation variables. When compared with molecular testing, DeepHRD classified 1.8- to 3.1-fold more patients with HRD, which exhibited better OS in high-grade serous ovarian cancer and platinum-specific PFS in metastatic breast cancer.

[27] "Autosurv: interpretable deep learning framework for cancer survival analysis incorporating clinical and multi-omics data" by Lindong Jiang, Chao Xu, Yuntong Bai, Anqi Liu, Yun Gong, Yu-Ping Wang, Hong-Wen Deng (2024)

Abstract: Accurate prognosis for cancer patients can provide critical information for optimizing treatment plans and improving life quality. Combining omics data and demographic/clinical information can offer a more comprehensive view of cancer prognosis than using omics or clinical data alone and can also reveal the underlying disease mechanisms at the molecular level. In this study, we developed and validated a deep learning framework to extract information from high-dimensional gene expression and miRNA expression data and conduct prognosis prediction for breast cancer and ovarian-cancer patients using multiple independent multi-omics datasets. Our model achieved significantly better prognosis prediction than the current machine learning and deep learning approaches in various settings. Moreover, an interpretation method was applied to tackle the "black-box" nature of deep neural networks and we identified features (i.e., genes, miRNA, demographic/clinical variables) that were important to distinguish predicted high- and low-risk patients. The significance of the identified features was partially supported by previous studies.

[28] "Deep learning infers clinically relevant protein levels and drug response in breast cancer from unannotated pathology images" by Hui Liu, Xiaodong Xie, Bin Wang (2024)

Abstract: The computational pathology has been demonstrated to effectively uncover tumor-related genomic alterations and transcriptomic patterns. Although proteomics has indeed shown great potential in the field of precision medicine, few studies have focused on the computational prediction of protein levels from pathology images. In this paper, we assume that deep learning-based pathological features imply the protein levels of tumor biomarkers that are indicative of prognosis and drug response. For this purpose, we propose wsi2rppa, a weakly supervised contrastive learning framework to infer the protein levels of tumor biomarkers from whole slide images (WSIs) in breast cancer. We first conducted contrastive learning-based pre-training on tessellated tiles to extract pathological features, which are then aggregated by attention pooling and adapted to downstream tasks. We conducted extensive evaluation experiments on the TCGA-BRCA cohort (1978 WSIs of 1093 patients with protein levels of 223 biomarkers) and the CPTAC-BRCA cohort (642 WSIs of 134 patients). The results showed that our method achieved state-of-the-art performance in tumor diagnostic tasks, and also performed well in predicting clinically relevant protein levels and drug response. To show the model interpretability, we spatially visualized the WSIs colored the tiles by their attention scores, and found that the regions with high scores were highly consistent with the tumor and necrotic regions annotated by a 10-year experienced pathologist. Moreover, spatial transcriptomic data further verified that the heatmap generated by attention scores agrees greatly with the spatial expression landscape of two typical tumor biomarker genes. In predicting the response to drug trastuzumab treatment, our method achieved a 0.79 AUC value which is much higher than the previous study reported 0.68. These findings showed the remarkable potential of computational pathology in the prediction of clinically relevant protein levels, drug response, and clinical outcomes.

[29] "Deep-Learning Model for Tumor-Type Prediction Using Targeted Clinical Genomic Sequencing Data" by Madison Darmofal, Shalabh Suman, Gurnit Atwal, Michael Toomey, Jie-Fu Chen, Jason C. Chang, Efsevia Vakiani, Anna M. Varghese, Anoop Balakrishnan Rema, Aijazuddin Syed, Nikolaus Schultz, Michael F. Berger,, Quaid Morris (null)

Abstract: [not available in provided text]

[30] "A multiomics analysis-assisted deep learning model identifies a macrophage-oriented module as a potential therapeutic target in colorectal cancer" by Xuanwen Bao, Qiong Li, Dong Chen, ..., Jianpeng Sheng, Jian Ruan, Peng Zhao (2024)

Abstract: [not available in the provided text]

[31] "Development of a deep learning model for cancer diagnosis by inspecting cell-free DNA end-motifs" by Hongru Shen, Meng Yang, Jilei Liu, Kexin Chen, Xiangchun Li (2024)

Abstract: Accurate discrimination between patients with and without cancer from cfDNA is crucial for early cancer diagnosis. Herein, we develop and validate a deep-learning-based model entitled end-motif inspection via transformer (EMIT) for discriminating individuals with and without cancer by learning feature representations from cfDNA end-motifs. EMIT is a self-supervised learning approach that models rankings of cfDNA end-motifs. We include 4606 samples subjected to different types of cfDNA sequencing to develop EIMIT, and subsequently evaluate classification performance of linear projections of EMIT on six datasets and an additional inhouse testing set encompassing whole-genome, whole-genome bisulfite and 5-hydroxymethylcytosine sequencing. The linear projection of representations from EMIT achieved area under the receiver operating curve (AUROC) values ranged from 0.895 (0.835–0.955) to 0.996 (0.994–0.997) across these six datasets, outperforming its baseline by significant margins. Additionally, we showed that linear projection of EMIT representations can achieve an AUROC of 0.962 (0.914–1.0) in identification of lung cancer on an independent testing set subjected to whole-exome sequencing. The findings of this study indicate that a transformer-based deep learning model can learn cancer-discriminative representations from cfDNA end-motifs. The representations of this deep learning model can be exploited for discriminating patients with and without cancer.

[32] "Aneuploidy as a driver of human cancer" by Eran Sdeor, Hajime Okada, Ron Saad, Tal Ben-Yishay, Uri Ben-David (2024)

Abstract: Aneuploidy, an abnormal chromosome composition, is a major contributor to cancer development and progression and an important determinant of cancer therapeutic responses and clinical outcomes. Despite being recognized as a hallmark of human cancer, the exact role of aneuploidy as a 'driver' of cancer is still largely unknown. Identifying the specific genetic elements that underlie the recurrence of common aneuploidies remains a major challenge of cancer genetics. In this Review, we discuss recurrent aneuploidies and their function as drivers of tumor development. We then delve into the context-dependent identification and functional characterization of the driver genes underlying driver aneuploidies and examine emerging strategies to uncover these driver genes using cancer genomics data and cancer models. Lastly, we explore opportunities for targeting driver aneuploidies in cancer by leveraging the functional consequences of these common genetic alterations.

[33] "Investigating the effects of artificial intelligence on the personalization of breast cancer management: a systematic study" by Solmaz Sohrabei, Hamid Moghaddasi, Azamossadat Hosseini, Seyed Jafar Ehsanzadeh (2024)

Abstract: Background Providing appropriate specialized treatment to the right patient at the right time is considered necessary in cancer management. Targeted therapy tailored to the genetic changes of each breast cancer patient is a desirable feature of precision oncology, which can not only reduce disease progression but also potentially increase patient survival. The use of artificial intelligence alongside precision oncology can help physicians by identifying and selecting more effective treatment factors for patients.

[34] "Deep learning approach for brain tumor classification using metaheuristic optimization with gene expression data" by Amol Avinash Joshi, Rabia Musheer Aziz (2024)

Abstract: This study addresses the critical challenge of accurately classifying brain tumors using artificial intelligence. Early detection is crucial, as untreated tumors can be fatal. Despite advances in AI, accurately classifying tumors remains a challenging task. To address this challenge, we propose a novel optimization approach called PSCS combined with deep learning for brain tumor classification. PSCS optimizes the classification process by improving Particle Swarm Optimization (PSO) exploitation using Cuckoo search (CS) algorithm. Next, classified gene expression data of brain tumor using Deep Learning (DL) to identify different groups or classes related to a particular tumor along with the PSCS optimization technique. The proposed optimization technique with DL achieves much better classification accuracy than other existing DL and Machine learning models with the different evaluation matrices such as Recall, Precision, F1-Score, and confusion matrix. This research contributes to AI-driven brain tumor diagnosis and classification, offering a promising solution for improved patient outcomes.

[35] "A panoptic segmentation dataset and deep-learning approach for explainable scoring of tumor-infiltrating lymphocytes" by Shangke Liu, Mohamed Amgad, Deeptej More, Muhammad A. Rathore, Roberto Salgado, Lee A. D. Cooper (2024)

Abstract: Tumor-Infiltrating Lymphocytes (TILs) have strong prognostic and predictive value in breast cancer, but their visual assessment is subjective. To improve reproducibility, the International Immuno-oncology Working Group recently released recommendations for the computational assessment of TILs that build on visual scoring guidelines. However, existing resources do not adequately address these recommendations due to the lack of annotation datasets that enable joint, panoptic segmentation of tissue regions and cells. Moreover, existing deep-learning methods focus entirely on either tissue segmentation or cell nuclei detection, which complicates the process of TILs assessment by necessitating the use of multiple models and reconciling inconsistent predictions. We introduce PanopTILs, a region and cell-level annotation dataset containing 814,886 nuclei from 151 patients, openly accessible at:sites.google.com/view/panoptils. Using PanopTILs we developed MuTILs, a neural network optimized for assessing TILs in accordance with clinical recommendations. MuTILs is a concept bottleneck model designed to be interpretable and to encourage sensible predictions at multiple resolutions. Using a rigorous internal-external cross-validation procedure, MuTILs achieves an AUROC of 0.93 for lymphocyte detection and a DICE coefficient of 0.81 for tumor-associated stroma segmentation. Our computational score closely matched visual scores from 2 pathologists (Spearman R= 0.58– 0.61, p < 0.001). Moreover, computational TILs scores had a higher prognostic value than visual scores, independent of TNM stage and patient age. In conclusion, we introduce a comprehensive open data resource and a modeling approach for detailed mapping of the breast tumor microenvironment.

[36] "An overview of artificial intelligence in the field of genomics" by Khizra Maqsood, Hani Hagras, Nicolae Radu Zabet (2024)

Abstract: Artificial intelligence (AI) is revolutionizing many real-world applications in various domains. In the field of genomics, multiple traditional machine-learning approaches have been used to understand the dynamics of genetic data. These approaches provided acceptable predictions; however, these approaches are based on opaque-box AI algorithms which are not able to provide the needed transparency to the community. Recently, the field of explainable artificial intelligence has emerged to overcome the interpretation problem of opaque box models by aiming to provide complete transparency of the model and its prediction to the users especially in sensitive areas such as healthcare, finance, or security. This paper highlights the need for eXplainable Artificial Intelligence (XAI) in the field of genomics and how the understanding of genomic regions, specifically the non-coding regulatory region of genomes (i.e., enhancers), can help uncover underlying molecular principles of disease states, in particular cancer in humans.

[37] "Precision oncology: Using cancer genomics for targeted therapy advancements" by Cigir Biray Avci, Bakiye Goker Bagca, Behrouz Shademan, Leila Sabour Takanlou, Maryam Sabour Takanlou, Alireza Nourazarian (2025)

Abstract: Cancer genomics plays a crucial role in oncology by enhancing our understanding of how genes drive cancer and facilitating the development of improved treatments. This field meticulously examines various cancers' genetic makeup through various methodologies, leading to groundbreaking discoveries. Innovative tools such as rapid gene sequencing, single-cell studies, spatial gene mapping, epigenetic analysis, liquid biopsies, and computational modeling have significantly progressed the field. These techniques uncover genetic alterations, tumor heterogeneity, and the evolutionary dynamics of cancers. Genetic abnormalities and molecular markers that initiate and propagate distinct cancer types are classified according to tumor type. The integration of precision medicine with cancer genomics emphasizes the significance of utilizing genetic data in treatment decision-making, enabling personalized care and enhancing patient outcomes. Critical topics in cancer genomics encompass tumor diversity, alterations in non-coding DNA, epigenetic modifications, cancer-specific proteins, metabolic changes, and the impact of inherited genes on cancer risk.

[38] "Synthetic augmentation of cancer cell line multi-omic datasets using unsupervised deep learning" by Zhaoxiang Cai, Sofia Apolinário, Ana R. Baião, Clare Pacini, Miguel D. Sousa, Susana Vinga, Roger R. Reddel, Phillip J. Robinson, Mathew J. Garnett, Qing Zhong, Emanuel Gonçalves (2024)

Abstract: Integrating diverse types of biological data is essential for a holistic understanding of cancer biology, yet it remains challenging due to data heterogeneity, complexity, and sparsity. Addressing this, our study introduces an unsupervised deep learning model, MOSA (Multi-Omic Synthetic Augmentation), specifically designed to integrate and augment the Cancer Dependency Map (DepMap). Harnessing orthogonal multi-omic information, this model successfully generates molecular and phenotypic profiles, resulting in an increase of 32.7% in the number of multi-omic profiles and thereby generating a complete DepMap for 1523 cancer cell lines. The synthetically enhanced data increases statistical power, uncovering less studied mechanisms associated with drug resistance, and refines the identification of genetic associations and clustering of cancer cell lines. By applying SHapley Additive exPlanations (SHAP) for model interpretation, MOSA reveals multi-omic features essential for cell clustering and biomarker identification related to drug and gene dependencies. This understanding is crucial for developing much-needed effective strategies to prioritize cancer targets.

[39] "Survival prediction of glioblastoma patients using machine learning and deep learning: a systematic review" by Roya Poursaeed, Mohsen Mohammadzadeh, Ali Asghar Safaei (2024)

Abstract: Glioblastoma Multiforme (GBM), classified as a grade IV glioma by the World Health Organization (WHO), is a prevalent and notably aggressive form of brain tumor derived from glial cells. It stands as one of the most severe forms of primary brain cancer in humans. The median survival time of GBM patients is only 12–15 months, making it the most lethal type of brain tumor. Every year, about 200,000 people worldwide succumb to this disease. GBM is also highly heterogeneous, meaning that its characteristics and behavior vary widely among different patients. This leads to different outcomes and survival times for each individual. Predicting the survival of GBM patients accurately can have multiple benefits. It can enable optimal and personalized treatment planning based on the patient's condition and prognosis. It can also support the patients and their families to cope with the possible outcomes and make informed decisions about their care and quality of life. Furthermore, it can assist the researchers and scientists to discover the most relevant biomarkers, features, and mechanisms of the disease and to design more effective and personalized therapies. Artificial intelligence methods, such as machine learning and deep learning, have been widely applied to survival prediction in various fields, such as breast cancer, lung cancer, gastric cancer, cervical cancer, liver cancer, prostate cancer, and covid 19. This systematic review summarizes the current state-of-the-art methods for predicting glioblastoma survival using different types of input data, such as clinical features, molecular markers, imaging features, radiomics features, omics data or a combination of them. Following PRISMA guidelines, we searched databases from 2015 to 2024, reviewing 107 articles meeting our criteria. We analyzed the data sources, methods, performance metrics and outcomes of the studies. We found that random forest was the most popular method, and a combination of radiomics and clinical data was the most common input data.

[40] "Deep learning and machine learning approaches to classify stomach distant metastatic tumors using DNA methylation profiles" by Jing Shi, Ying Chen, Ying Wang (2024)

Abstract: Distant metastasis of cancer is a significant contributor to cancer-related complications, and early identification of unidentified stomach adenocarcinoma is crucial for a positive prognosis. Changes in DNA methylation are being increasingly recognized as a crucial factor in predicting cancer progression. Within this research, we developed machine learning and deep learning models for distinguishing distant metastasis in samples of stomach adenocarcinoma based on DNA methylation profile. Employing deep neural networks (DNN), support vector machines (SVM), random forest (RF), Naive Bayes (NB) and decision tree (DT), and models for forecasting distant metastasis in stomach adenocarcinoma. The results show that the performance of DNN is better than that of other models, AUC and AUPR achieving 99.9 % and 99.5 % respectively. Additionally, a weighted random sampling technique was utilized to address the issue of imbalanced datasets, enabling the identification of crucial methylation markers associated with functionally significant genes in stomach distant metastasis tumors with greater performance.

[41] "Deep Learning of radiology-genomics integration for computational oncology: A mini review" by Feng-ao Wang, Yixue Li, Tao Zeng (2024)

Abstract: In the field of computational oncology, patient status is often assessed using radiology-genomics, which includes two key technologies and data, such as radiology and genomics. Recent advances in deep learning have facilitated the integration of radiology-genomics data, and even new omics data, significantly improving the robustness and accuracy of clinical predictions. These factors are driving artificial intelligence (AI) closer to practical clinical applications. In particular, deep learning models are crucial in identifying new radiology-genomics biomarkers and therapeutic targets, supported by explainable AI (xAI) methods. This review focuses on recent developments in deep learning for radiology-genomics integration, highlights current challenges, and outlines some research directions for multimodal integration and biomarker discovery of radiology-genomics or radiology-omics that are urgently needed in computational oncology.

[42] "The power of deep learning in simplifying feature selection for hepatocellular carcinoma: a review" by Ghada Mostafa, Hamdi Mahmoud, Tarek Abd El-Hafeez, Mohamed E.ElAraby (2024)

Abstract: Background Hepatocellular Carcinoma (HCC) is a highly aggressive, prevalent, and deadly type of liver cancer. With the advent of deep learning techniques, significant advancements have been made in simplifying and optimizing the feature selection process. Objective Our scoping review presents an overview of the various deep learning models and algorithms utilized to address feature selection for HCC. The paper highlights the strengths and limitations of each approach, along with their potential applications in clinical practice. Additionally, it discusses the benefits of using deep learning to identify relevant features and their impact on the accuracy and efficiency of diagnosis, prognosis, and treatment of HCC. Design The review encompasses a comprehensive analysis of the research conducted in the past few years, focusing on the methodologies, datasets, and evaluation metrics adopted by different studies. The paper aims to identify the key trends and advancements in the field, shedding light on the promising areas for future research and development. Results The findings of this review indicate that deep learning techniques have shown promising results in simplifying feature selection for HCC. By leveraging large-scale datasets and advanced neural network architectures, these methods have demonstrated improved accuracy and robustness in identifying predictive features. Conclusions We analyze published studies to reveal the state-of-the-art HCC prediction and showcase how deep learning can boost accuracy and decrease false positives. But we also acknowledge the challenges that remain in translating this potential into clinical reality.

[43] "Histopathologic image–based deep learning classifier for predicting platinum-based treatment responses in high-grade serous ovarian cancer" by Byungsoo Ahn, Damin Moon, Hyun-Soo Kim, Chung Lee, Nam Hoon Cho, Heung-Kook Choi, Dongmin Kim, Jung-Yun Lee, Eun Ji Nam, Dongju Won, Hee Jun An, Sun Young Kwon, Su-Jin Shin, Hye Ra Jung, Dohee Kwon, Heejung Park, Milim Kim, Yoon Jin Cha, Hyunjin Park, Yangkyu Lee, Songmi Noh, Yong-Moon Lee, Sung-Eun Choi, Ji Min Kim, Sun Hee Sung, Eunhyang Park (2024)

Abstract: Platinum-based chemotherapy is the cornerstone treatment for female high-grade serous ovarian carcinoma (HGSOC), but choosing an appropriate treatment for patients hinges on their responsiveness to it. Currently, no available biomarkers can promptly predict responses to platinum-based treatment. Therefore, we developed the Pathologic Risk Classifier for HGSOC (PathoRiCH), a histopathologic image–based classifier. PathoRiCH was trained on an in-house cohort (n = 394) and validated on two independent external cohorts (n = 284 and n = 136). The PathoRiCH-predicted favorable and poor response groups show significantly different platinum-free intervals in all three cohorts. Combining PathoRiCH with molecular biomarkers provides an even more powerful tool for the risk stratification of patients. The decisions of PathoRiCH are explained through visualization and a transcriptomic analysis, which bolster the reliability of our model's decisions. PathoRiCH exhibits better predictive performance than current molecular biomarkers. PathoRiCH will provide a solid foundation for developing an innovative tool to transform the current diagnostic pipeline for HGSOC.

[44] "A deep learning model of tumor cell architecture elucidates response and resistance to CDK4/6 inhibitors" by Sungjoon Park, Erica Silva, Akshat Singhal, Marcus R. Kelly, Kate Licon, Isabella Panagiotou, Catalina Fogg, Samson Fong, John J. Y. Lee, Xiaoyu Zhao, Robin Bachelder, Barbara A. Parker, Kay T. Yeung, Trey Ideker (2024)

Abstract: Cyclin-dependent kinase 4 and 6 inhibitors (CDK4/6is) have revolutionized breast cancer therapy. However, <50% of patients have an objective response, and nearly all patients develop resistance during therapy. To elucidate the underlying mechanisms, we constructed an interpretable deep learning model of the response to palbociclib, a CDK4/6i, based on a reference map of multiprotein assemblies in cancer. The model identifies eight core assemblies that integrate rare and common alterations across 90 genes to stratify palbociclib-sensitive versus palbociclib-resistant cell lines. Predictions translate to patients and patient-derived xenografts, whereas single-gene biomarkers do not. Most predictive assemblies can be shown by CRISPR–Cas9 genetic disruption to regulate the CDK4/6i response. Validated assemblies relate to cell-cycle control, growth factor signaling and a histone regulatory complex that we show promotes S-phase entry through the activation of the histone modifiers KAT6A and TBL1XR1 and the transcription factor RUNX1. This study enables an integrated assessment of how a tumor's genetic profile modulates CDK4/6i resistance.

[45] "Advances in spatial transcriptomics and its applications in cancer research" by Yang Jin, Yuanli Zuo, Gang Li, Wenrong Liu, Yitong Pan, Ting Fan, Xin Fu, Xiaojun Yao, Yong Peng (2024)

Abstract: Malignant tumors have increasing morbidity and high mortality, and their occurrence and development is a complicate process. The development of sequencing technologies enabled us to gain a better understanding of the underlying genetic and molecular mechanisms in tumors. In recent years, the spatial transcriptomics sequencing technologies have been developed rapidly and allow the quantification and illustration of gene expression in the spatial context of tissues. Compared with the traditional transcriptomics technologies, spatial transcriptomics technologies not only detect gene expression levels in cells, but also inform the spatial location of genes within tissues, cell composition of biological tissues, and interaction between cells. Here we summarize the development of spatial transcriptomics technologies, spatial transcriptomics tools and its application in cancer research. We also discuss the limitations and challenges of current spatial transcriptomics approaches, as well as future development and prospects.

[46] "From cancer big data to treatment: Artificial intelligence in cancer research" by Danishuddin, Shawez Khan, Jong Joo Kim (2024)

Abstract: In recent years, developing the idea of "cancer big data" has emerged as a result of the significant expansion of various fields such as clinical research, genomics, proteomics and public health records. Advances in omics technologies are making a significant contribution to cancer big data in biomedicine and disease diagnosis. The increasingly availability of extensive cancer big data has set the stage for the development of multimodal artificial intelligence (AI) frameworks. These frameworks aim to analyze high-dimensional multi-omics data, extracting meaningful information that is challenging to obtain manually. Although interpretability and data quality remain critical challenges, these methods hold great promise for advancing our understanding of cancer biology and improving patient care and clinical outcomes. Here, we provide an overview of cancer big data and explore the applications of both traditional machine learning and deep learning approaches in cancer genomic and proteomic studies. We briefly discuss the challenges and potential of AI techniques in the integrated analysis of omics data, as well as the future direction of personalized treatment options in cancer.

[47] "A machine learning and deep learning-based integrated multi-omics technique for leukemia prediction" by Erum Yousef Abbasi, Zhongliang Deng, Qasim Ali, Adil Khan, Asadullah Shaikh, Mana Saleh Al Reshan, Adel Sulaiman, Hani Alshahrani (2024)

Abstract: In recent years, scientific data on cancer has expanded, providing potential for a better understanding of malignancies and improved tailored care. Advances in Artificial Intelligence (AI) processing power and algorithmic development position Machine Learning (ML) and Deep Learning (DL) as crucial players in predicting Leukemia, a blood cancer, using integrated multi-omics technology. However, realizing these goals demands novel approaches to harness this data deluge. This study introduces a novel Leukemia diagnosis approach, analyzing multi-omics data for accuracy using ML and DL algorithms. ML techniques, including Random Forest (RF), Naive Bayes (NB), Decision Tree (DT), Logistic Regression (LR), Gradient Boosting (GB), and DL methods such as Recurrent Neural Networks (RNN) and Feedforward Neural Networks (FNN) are compared. GB achieved 97 % accuracy in ML, while RNN outperformed by achieving 98 % accuracy in DL. This approach filters unclassified data effectively, demonstrating the significance of DL for leukemia prediction. The testing validation was based on 17 different features such as patient age, sex, mutation type, treatment methods, chromosomes, and others. Our study compares ML and DL techniques and chooses the best technique that gives optimum results. The study emphasizes the implications of high-throughput technology in healthcare, offering improved patient care.

**Generated Text:**

| The exponential growth of high-throughput omics technologies has fundamentally transformed cancer research by enabling the generation of unprecedented volumes of multi-dimensional molecular data. These technological advances have produced comprehensive datasets encompassing genomic sequences, transcriptomic profiles, proteomic signatures, and epigenetic modifications across diverse cancer types, creating new opportunities for understanding tumor biology and developing personalized therapeutic strategies (Hwang et al., 2024; Yang et al., 2025). The accumulation of these large-scale cancer omics datasets, often referred to as "cancer big data," presents both remarkable opportunities and significant analytical challenges that traditional computational methods struggle to address effectively (Danishuddin et al., 2024). In this context, artificial intelligence, particularly deep learning approaches, has emerged as a transformative force in extracting clinically actionable insights from complex, high-dimensional cancer datasets that would otherwise remain computationally intractable.  Deep learning technologies have demonstrated remarkable capabilities in analyzing diverse data modalities within cancer genomics, ranging from histopathological images to multi-omics molecular profiles. In histopathology, deep learning models can predict molecular alterations, treatment responses, and patient prognosis directly from hematoxylin and eosin stained whole-slide images, achieving area under the curve values exceeding 0.75 across multiple cancer types (El Nahhas et al., 2025; Rakaee et al., 2025). These computational pathology approaches represent a paradigm shift from traditional manual image analysis, enabling the extraction of quantitative features and patterns that may be imperceptible to human pathologists (Jiang et al., 2024). Beyond histopathology, deep learning has shown substantial promise in integrating multi-omics data to characterize tumor heterogeneity, identify therapeutic targets, and stratify patients for personalized treatment strategies (Shao et al., 2024; Yuan et al., 2024). Multimodal deep learning frameworks that simultaneously process genomic, transcriptomic, and proteomic information have demonstrated superior performance compared to single-modality approaches in cancer subtype classification and survival prediction tasks (Mohamed & Ezugwu, 2024).  Despite these technological advances, the clinical translation of artificial intelligence-based cancer genomics analysis faces persistent challenges that must be addressed to realize its full potential in precision oncology. Model interpretability remains a critical concern, as many deep learning architectures function as "black boxes" that provide predictions without transparent reasoning, complicating their acceptance in clinical decision-making workflows (Ramon et al., 2024). Furthermore, robust validation across diverse patient populations and healthcare settings is essential to ensure that AI models generalize beyond their training datasets and perform reliably in real-world clinical environments (Unger & Kather, 2024). The integration of AI-based predictions with existing medical workflows, electronic health record systems, and clinical guidelines presents additional practical obstacles that require careful consideration of institutional infrastructure, regulatory requirements, and clinician acceptance.  In this review, we examine recent advances in artificial intelligence-powered cancer genomics analysis, focusing on applications in histopathology image analysis, multi-omics data integration, and spatial transcriptomics. We assess the current state of achievements in biomarker discovery, therapeutic target identification, and treatment response prediction, while critically evaluating the limitations and challenges that impede clinical implementation. Finally, we discuss future directions for developing interpretable, clinically validated AI solutions that can be seamlessly integrated into precision oncology practices. |
| --- |
